# Methodological workflow for assessing heterogeneity in immature organic-rich carbonate reservoirs

**DOI:** 10.1016/j.mex.2026.103875

**Published:** 2026-03-20

**Authors:** Samer Aljurf, Israa S. Abu-Mahfouz, Septriandi Chan

**Affiliations:** a Department of Geosciences, King Fahd University of Petroleum and Minerals, Dhahran, Saudi Arabia; b Center for Integrative Petroleum Research, King Fahd University of Petroleum and Minerals, Dhahran, Saudi Arabia; c National Energy Services Reunited, NESR, Dhahran, Saudi Arabia

**Keywords:** Carbonate reservoirs, Heterogeneity, Lithofacies, Chemofacies, Ultrasonic velocities, Scratch test, Micro-rebound test

## Abstract

Understanding the vertical and lateral heterogeneity of immature organic-rich carbonate reservoirs is essential for predicting reservoir quality, maintaining wellbore stability, and identifying sweet-spot intervals in unconventional systems. Although geological, geochemical, petrophysical, and mechanical techniques are extensively employed for rock characterization, they are frequently applied in isolation, which can hinder a systematic assessment of variability. This paper introduces a structured, reproducible workflow that integrates lithological description, multivariate geochemical classification, continuous petrophysical profiling, and non-destructive mechanical testing into a unified framework for evaluating heterogeneity. The methodological innovation resides not in the individual analytical techniques but in their sequential integration into a facies-guided workflow that seamlessly links continuous non-destructive measurements with targeted laboratory analyses and predictive data integration. The workflow combines lithological descriptions, rock classification using unsupervised machine learning methods such as silhouette analysis, principal component analysis (PCA), and hierarchical clustering on principal component analysis (HCPC), bulk geochemistry, petrophysical profiling, non-destructive rock mechanical testing, and integrated data interpretation. This integrated method allows for accurate characterization of these complex reservoirs, supporting a systematic assessment of their heterogeneity, quality, and petrophysical and mechanical behavior. Continuous measurements are first used to capture spatial variability along the core interval, after which representative samples from identified facies domains are selected for laboratory analyses, including mineralogy, organic geochemistry, and petrophysical measurements. The integration of continuous and discrete datasets enables the evaluation of key properties across the entire core interval and systematic identification of heterogeneous zones. Lateral heterogeneity is identified by comparing lithofacies transitions, chemofacies shifts, and coordinated changes in porosity, velocity (Vp), total organic carbon (TOC), and strength (UCS) and hardness (HLD) logs. Moreover, the proposed workflow provides a clear methodological pathway for assessing vertical and lateral heterogeneity in immature organic-rich carbonate systems and can be adapted to unconventional hydrocarbon reservoirs, CO_2_ storage formations, geothermal systems, and mineral exploration studies.

## Highlights

• This comprehensive and integrated workflow is applicable across diverse fields, including unconventional hydrocarbon reservoirs, CO₂ storage, geothermal systems, and mineral resources exploration.
• It combines lithological, geochemical, petrophysical, and rock mechanical data to generate continuous, high-resolution profiles along the core interval, capturing rock heterogeneity.
• The approach is particularly effective for immature organic-rich carbonate reservoirs, improving understanding of their complex nature.

## Graphical abstract

Summited separately

**Figure:**
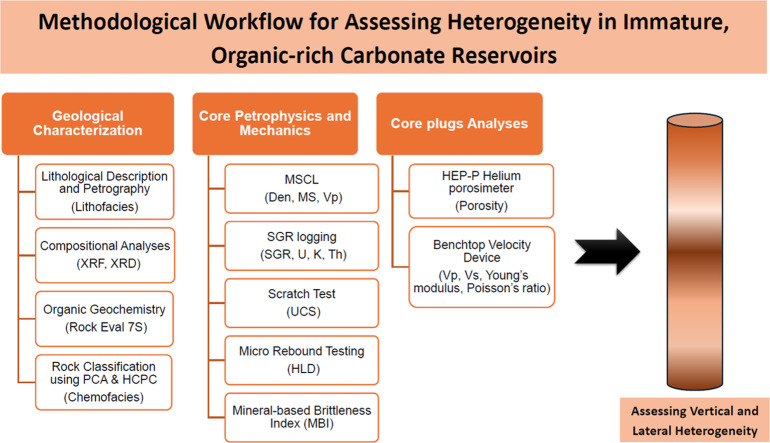

**Table:** Specifications table.

**Subject area**	Earth and Planetary Sciences
**More specific subject area**	Integrated rock characterization workflow
**Name of your method**	Assessing the vertical and lateral heterogeneity in immature organic-rich carbonate reservoirs
**Name and reference of original method**	Dunham, R.J., 1962. Classification of carbonate rocks according to depositional texture. Jolliffe, I., 2011. Principal component analysis. In International encyclopedia of statistical science (pp. 1094–1096). Springer, Berlin, Heidelberg. Husson, F., Lê, S. and Pagès, J., 2011. Exploratory multivariate analysis by example using R (Vol. 15, pp. 1–60). Boca Raton: CRC press. Germay, C., Richard, T., Mappanyompa, E., Lindsay, C., Kitching, D. and Khaksar, A., 2015. The continuous-scratch profile: a high-resolution strength log for geomechanical and petrophysical characterization of rocks. SPE Reservoir Evaluation & Engineering, 18(03), pp.432–440. Leeb, D., 1979. Dynamic hardness testing of metallic materials. NDT International, 12(6), pp.274–278. Glorioso, J. C. and Rattia, A. J., 2011. Petrophysical Characterization of Unconventional Reservoirs. SPE Middle East Oil and Gas Show and Conference, SPE-144,044-MS.
**Resource availability**	N.A

## Background

Immature organic-rich carbonate reservoirs are high-potential unconventional reservoirs due to their early maturity and hydrocarbon generation potential. However, they encounter lithological, geochemical, petrophysical, and mechanical heterogeneity. Without a thorough understanding of this heterogeneity, developing and producing from them becomes a challenging task. This study aims to assess and understand this heterogeneity by integrating lithological, geochemical, petrophysical, and rock mechanical methods into a comprehensive and unified workflow.

Although the individual analytical techniques used in this study are well established, their integration into a structured, high-resolution workflow constitutes the methodological contribution of this study. The workflow is designed to first capture continuous variability along the core through non-destructive measurements, then objectively classify compositional domains through lithofacies and chemofacies analysis, followed by facies-guided destructive laboratory sampling. Finally, continuous and discrete datasets are integrated to evaluate heterogeneity along the entire core interval. This systematic integration enables heterogeneity in organic-rich carbonates to be quantified more effectively than approaches that rely on isolated measurements.

This workflow aids in assessing and identifying vertical heterogeneity and key factors influencing property variability in the immature, Upper Cretaceous organic-rich carbonate source rocks of the of Jordan. In addition to characterizing these immature organic-rich carbonate reservoirs, it offers a standardized method for evaluating rock heterogeneity. This approach directly impacts reservoir modeling, well completion, and development planning in unconventional carbonate systems. Additionally, this workflow shows potential for application in geothermal energy, CO2 storage, and mineral resource exploration.

The novelty of this workflow lies in the systematic integration of geological, geochemical, petrophysical, and mechanical datasets within a high-resolution framework that first characterizes continuous variability along the core, thereby informing targeted laboratory analyses and predictive data propagation.

## Method details

**Fig. 1:**
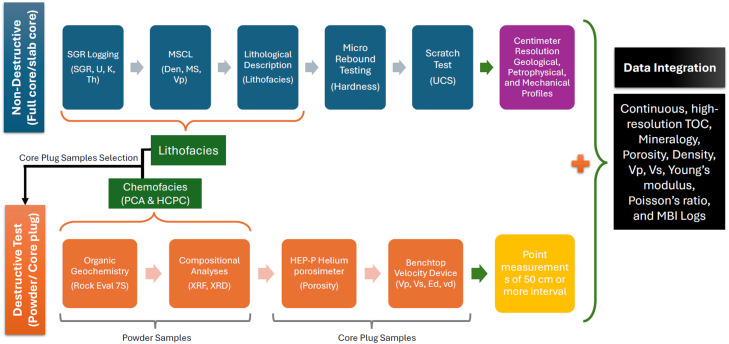
The integrated methodological workflow for characterizing and assessing the heterogeneity of immature carbonate reservoirs.

This study presents an integrated methodological workflow designed to systematically characterize heterogeneity in immature organic-rich carbonate reservoirs (Fig. 1). While the analytical techniques used in this study are individually routine, the novelty of the approach lies in their integration into a structured workflow that links continuous non-destructive measurements with targeted destructive laboratory analyses and integrated data interpretation.

The workflow consists of four sequential steps:

1 Non-destructive high-resolution characterization.
2 Identification of chemofacies.
3 Destructive analyses.
4 Data integration and predictive modeling.

### Step 1. Non-destructive high-resolution characterization

Prior to destructive sampling, the entire core interval undergoes a series of non-destructive measurements to generate continuous, high-resolution datasets. These measurements maintain spatial variability along the core and detect subtle heterogeneity that may be overlooked by conventional point-based measurement techniques.

Lithological description is performed on slabbed core surfaces through visual observations and using a 10 × hand lens. Lithofacies are classified based on the Dunham carbonate classification system [1]. Petrographic analysis of thin sections (∼30 µm thick) is also conducted to assess mineral composition, sedimentary textures, fossil content, and diagenetic features.

Continuous petrophysical data are acquired using a Multi-Sensor Core Logger (MSCL), which provides high-resolution profiles of bulk density, compressional-wave velocity (Vp), and magnetic susceptibility (MS) along the core length. These are measured at 5 cm intervals. In addition, spectral gamma-ray (SGR) analyses are performed to quantify the concentrations of uranium, thorium, and potassium and to obtain the SGR.

Rock mechanical variability is assessed through non-destructive testing methods. Hardness is measured with a micro-rebound testing device (Proceq Equotip Bambino2). Leeb Hardness is tested every 5 cm. Calibration with a reference standard (D-block) ensures accurate results.

The mineral-based brittleness index (MBI) was derived from mineralogical data using the equation proposed by Glorioso and Rattia [2].

(1) $$MBI_{GR}=\frac{Qz+Carb}{Qz+Carb+Clay+TOC}$$

Where Qz is quartz percentage (%), Carb is carbonate percentage (Ca% + Mg%), Clay percentage (%), and TOC percentage (%).

Continuous strength profiles are obtained via scratch testing with the Epslog Wombat device to measure the Intrinsic Specific Energy (ISE) from the core slabbed surfaces. The ISE readings are then translated into unconfined compressive strength (UCS) to generate an accurate strength log.

### Step 2. Identification of chemofacies

The continuous high-resolution dataset is then analyzed using multivariate statistical techniques to identify chemofacies. This step allows the heterogeneity of the organic-rich carbonate to be objectively classified based on mineralogical and geochemical variations along the core.

Principal component analysis (PCA) is first applied to reduce dimensionality and identify dominant geochemical trends within the dataset. Hierarchical clustering on principal components (HCPC) is subsequently used to group samples into clusters with similar geochemical signatures. These clusters represent chemofacies reflecting variations in mineralogical composition, organic matter content, depositional conditions, and diagenetic alteration. This classification is more reliable and less subjective than lithofacies.

### Step 3. Destructive analyses

Representative samples from each identified lithofacies and chemofacies are then selected for destructive laboratory analyses (e.g., Rock-Eval pyrolysis, XRD, XRF, Porosity). This ensures that laboratory testing captures the full variability of the system rather than relying on arbitrary sampling.

To better understand variability in minerals and organic material, powdered samples with particle sizes (< 50 µm) should be systematically collected at intervals of at least 50 cm vertically to capture vertical heterogeneity. Mineral contents are determined using XRD, while XRF is employed to analyze both major and trace elements.

Additionally, Rock-Eval 7S pyrolysis was performed to measure total organic carbon (TOC), hydrogen index (HI), sulfur content (TS), Tmax, and other maturity parameters (S_1_, S_2_, and S_3_). This helps in determining the quality and maturity level of the kerogen. This analysis was performed using approximately 30–50 mg of powdered sample in an inert nitrogen atmosphere. The temperature program consisted of an initial isothermal stage at 300 °C for the measurement of free hydrocarbons (S_1_), followed by a programmed heating to 650 °C at a rate of 25 °C/min to assess the residual hydrocarbon potential (S_2_) and carbon dioxide evolution (S_3_). Tmax was defined as the temperature at which the S_2_ peak was maximum.

Porosity was measured using a HEP-P Helium porosimeter instrument on extracted core plug samples with a 1-inch diameter. The variability in porosity values can also indicate changes in the depositional environment or conditions that affect the inherited properties of the rocks.

Ultrasonic velocities were measured using a benchtop velocity measurement device (in our study, we used the NER Autolab benchtop velocity device from New England Research Company). Measurements were performed at ambient pressure using 1-inch core plugs. Travel times data were converted to compressional wave (Vp) and shear wave (Vs) based on the measured length of the plugs, and from these velocities, the dynamic Young’s modulus and Poisson’s ratio were subsequently calculated.

### Step 4. Data integration and predictive modeling

The final step of the workflow involves integrating continuous high-resolution measurements with discrete laboratory datasets to evaluate heterogeneity across the entire core interval. All datasets are depth-matched and combined into a single database to ensure consistency in stratigraphic alignment. By correlating continuous petrophysical and mechanical profiles with facies classification and laboratory measurements, a systematic evaluation of variability in lithology, mineral composition, organic content, and mechanical properties is conducted. Intervals where facies transitions align with corresponding changes in petrophysical and mechanical properties are interpreted as heterogenous zones. This integrated approach provides a robust framework for quantifying heterogeneity in organic-rich carbonate systems and ensures that variability is assessed through multiple independent datasets rather than relying on a single parameter.

## Method summary

### Sample acquisition and preparation

  • Begin by acquiring a vertical core sample by drilling into the target formation and slabbing the core for detailed observation.
  • Collect representative samples at regular intervals (e.g., 50 cm) for geochemical analyses.
  • Prepare representative thin sections from each lithofacies and chemofacies (∼30 µm) for petrographic imaging.
  • Prepare core plug samples with a 1-inch diameter for porosity and ultrasonic velocity measurements. Samples should be collected at fixed vertical intervals (e.g., 50 cm). Additional samples may be obtained across visually identifiable lithological boundaries to more effectively capture abrupt changes in rock properties and enhance the characterization of heterogeneity.

### Lithological and petrographic characterization

  • Describe the core slabs by naked eye and with a 10X lens according to Dunham’s [1] classification, documenting lithology, color, texture, lamination, and fossil content.
  • Examine the thin sections with optical microscopy to assess lithofacies, mineral components, diagenetic features, fossil assemblages, and fracture fillings.

### Bulk inorganic and organic geochemistry

  • Analyze powdered samples with X-ray diffraction (XRD) for mineralogy and X-ray fluorescence (XRF) for bulk elemental composition.
  • Assess the organic richness, maturation level, and kerogen type using Rock-Eval 7S pyrolysis (S1, S2, TOC, TS, HI, Tmax).

### Unsupervised machine learning (chemofacies)

  • Use multivariate statistical analysis to combine geochemical datasets. First, conduct Silhouette analysis to determine the optimal number of clusters.
  • Perform PCA to reduce dimensionality and emphasize main geochemical trends, while using HCPC to cluster samples into distinct chemofacies.

### Petrophysical logging

  • Perform non-destructive core scale measurements with a Multi-Sensor Core Logger (MSCL), providing continuous and high-resolution bulk density, Vp, and MS data.
  • Conduct spectral gamma-ray (SGR) logging to determine concentrations of uranium, thorium, potassium, and SGR.
  • Measure the porosity of the extracted core plugs using the HEP-P Helium porosimeter.

### Rock mechanical profiling

  • Determine the strength (UCS) with the scratch testing device.
  • Measure the rock hardness with the micro-rebound testing device (Bambino 2) along the core interval.
  • Calculate the mineral-based brittleness index (MBI_GR) using the equation from Glorioso and Rattia [2] (Eq. (1)).
  • Measure the compressional and shear wave velocities at ambient pressure for core plug samples using the NER benchtop velocity device, then calculate the dynamic Poisson’s ratio and Young’s modulus. Each core plug sample should be carefully oriented to ensure full contact with both transducers, thereby optimizing ultrasonic wave transmission through the sample.

### Data integration and heterogeneity assessment

Heterogeneity is defined operationally as the observable vertical or lateral variability in lithofacies or chemofacies, as well as in rock properties, which reflect variations in depositional texture, mineralogical composition, organic content, petrophysical attributes, and mechanical behavior. A rock interval is classified as heterogeneous when multiple independent datasets exhibit coordinated variations at the same depth. This cross-validation among geological, geochemical, petrophysical, and mechanical datasets mitigates interpretational ambiguity and enhances the robustness of heterogeneity characterization.

All datasets should be systematically depth-matched and integrated into a comprehensive database. Followed by analyzing the vertical and lateral variability across the integrated dataset to assess heterogeneity. Instead of relying on a single parameter, variability is now assessed by comparing lithofacies/chemofacies and the associated petrophysical and mechanical property trends with depth. The workflow is executed in three sequential steps:

1. Domain-specific characterization

Each dataset needs to be interpreted first independently:

• Define lithofacies using Dunham’s classification and petrographic observations.
• Analyze geochemical datasets using PCA and HCPC to define chemofacies.
• Plot petrophysical and mechanical properties as continuous vertical profiles.

The number of identified lithofacies types and chemofacies clusters reflects compositional and textural variability within the succession. Similarly, the wide range (high standard deviation) and fluctuations in petrophysical and mechanical property values provide insight into intrinsic physical and mechanical contrasts within the rock interval, reflecting heterogeneity within the dataset.

2. Vertical heterogeneity assessment

Vertical heterogeneity can be identified by observing changes with depth in:

• Lithofacies transitions
• Chemofacies cluster shifts
• Variations in porosity, Vp, TOC, and mechanical strength

The interbedding of lithofacies or chemofacies in the section generates cm- to m-scale compositional heterogeneity in the vertical dimension. Moreover, changes in petrophysical and mechanical properties, along with depth, effectively captured vertical heterogeneity within the studied samples. Intervals where facies transitions coincide with noticeable shifts in petrophysical and mechanical properties were interpreted as vertically heterogeneous.

3. Lateral heterogeneity assessment

Lateral heterogeneity is evaluated through well-to-well correlation of lithofacies, chemofacies, and associated property trends. Differences in facies proportions, cluster distributions, and rock-property patterns among wells are used to delineate spatial variability within the reservoir.

This structured integration approach provides a reproducible framework for characterizing heterogeneity in immature organic-rich carbonate systems by linking compositional domains directly to petrophysical and mechanical variability. By integrating multiple independent datasets, the workflow enhances interpretative confidence and allows heterogeneity to be assessed systematically rather than being inferred qualitatively, thereby reducing reliance on a single dataset.

In our recent study (Aljurf et al., [3]), heterogeneity is characterized as scale-dependent variability in lithological, geochemical, petrophysical, and mechanical properties observed along the depth profile. Rather than being inferred from a single parameter, heterogeneity is identified through the coordinated variability across multiple independent datasets. The data integration approach is founded upon three core principles:

1 Depth consistency: All datasets are aligned to a common stratigraphic framework to facilitate direct comparability.
2 Domain independence: Geological, geochemical, petrophysical, and mechanical datasets are initially interpreted independently to ascertain their intrinsic variability.
3 Cross-domain validation: Heterogeneity is confirmed only when variability is corroborated by more than one dataset.

### Application

  • The integrated workflow is used to reconstruct depositional architecture, environment, and conditions, aiding in creating accurate geological and reservoir models.
  • This workflow is used to assess vertical heterogeneity within a single well, while correlation between wells helps evaluate and determine heterogeneity both vertically and laterally within the studied reservoir.
  • Unlike approaches that depend on isolated measurements or averaged data, this workflow underscores the importance of depth-matched integration and facies-aware interpretation. By explicitly linking chemofacies clustering with mechanical and petrophysical variability, it mitigates the risk of oversimplifying reservoir heterogeneity during upscaling processes. Additionally, the approach recognizes that heterogeneity intensifies with increasing measurement resolution, thereby providing a structured framework to identify and contextualize such variability within immature organic-rich carbonate systems.
  • By explicitly linking facies classification with continuous petrophysical and mechanical measurements, the workflow reduces uncertainty associated with upscaling discrete laboratory data and provides a reproducible approach for evaluating reservoir heterogeneity.

### Method validation

Repeated petrophysical and mechanical tests showed deviations of <7%, indicating the high reproducibility of the core-scale testing method. This level of consistency ensures that the workflow can be reliably duplicated in other labs with standard analytical equipment.

### Limitations

The presented methodological workflow provides a comprehensive, structured, and systematic approach to assess the vertical and lateral heterogeneity of immature organic-rich carbonate reservoirs. However, this workflow required high-quality core samples and access to cutting-edge measurement devices, which may not be available at every institute.

## Ethics statements

None.

## CRediT authorship contribution statement

**Samer Aljurf:** Conceptualization, Methodology, Software, Validation, Formal analysis, Investigation, Visualization, Writing – original draft. **Israa S. Abu-Mahfouz:** Conceptualization, Methodology, Validation, Investigation, Resources, Writing – review & editing, Supervision, Project administration, Funding acquisition. **Septriandi Chan:** Methodology, Software, Validation, Resources, Writing – review & editing.

## Declaration of interests

The authors declare that they have no competing interests.

## Data Availability

Data will be made available on request.
